# Estrogens produced within the central amygdala inhibit varicella zoster‐induced orofacial pain

**DOI:** 10.1111/jne.70012

**Published:** 2025-02-27

**Authors:** Phillip Kramer, Lauren Nguyen, Paul R. Kinchington

**Affiliations:** ^1^ Department of Biomedical Sciences Texas A&M University College of Dentistry Dallas Texas USA; ^2^ Departments of Ophthalmology and of Molecular Microbiology and Genetics University of Pittsburgh Pittsburgh Pennsylvania USA

**Keywords:** amygdala, aromatase, estrogen, orofacial, pain

## Abstract

Varicella zoster virus (VZV) causes chicken pox, and reactivation of this virus later in life causes shingles. Previous work demonstrated that estrogens could reduce VZV‐induced orofacial pain and affect gene expression in the central amygdala. It is known that the central amygdala processes pain signals from the orofacial region and that estrogens produced by the enzyme aromatase within the central amygdala regulate neuronal function. Based on the previous studies, it was hypothesized estrogens produced within the central amygdala attenuate VZV‐induced orofacial pain. To address this hypothesis, male Long–Evans rats were implanted with cannulas terminating in the central amygdala. Through these cannulas, the aromatase inhibitor letrozole or estrogen receptor alpha (ERα) agonist, 4,4′,4″‐(4‐propyl‐[1*H*]‐pyrazole‐1,3,5‐triyl)*tris*phenol (PPT), was infused in the central amygdala. The whisker pad of each rat was injected with either MeWo cells or MeWo cells containing VZV. One week after VZV injection, letrozole or PPT was infused into the central amygdala, followed by measuring pain behavior, GABA release, and estradiol concentrations. Tissues in the orofacial pain pathway were isolated, and neuronal activity was quantitated by counting c‐Fos‐positive neurons. Letrozole significantly increased the pain response and decreased GABA release. Letrozole also decreased estradiol within the central amygdala. Infusion of PPT reduced pain and increased GABA release. Moreover, letrozole increased the number of active neurons in the lateral parabrachial nucleus and spinal trigeminal nucleus, while PPT reduced the number of active neurons in the trigeminal ganglia, lateral parabrachial nucleus, and spinal trigeminal nucleus. The results suggest aromatase‐derived estradiol interacts with ERα within the central amygdala to attenuate VZV‐induced pain by increasing GABA release and reducing neuronal activity in the pain pathway.

## 1 INTRODUCTION

Varicella zoster virus (VZV) results in chickenpox and remains latent in sensory nerve ganglia.^1^ Reactivation of the virus causes shingles^2^, ^3^ often manifesting as blisters, a dermatomal rash and pain.^4^ This pain is likely due to nerve damage^5^ to sensory nerve roots.^6^ Shingles is common in the United States^2^, ^7^ where one in three individuals develops shingles in their lifetime.^8^ Today, with current vaccines, VZV still results in 20% of patients developing chronic pain^8^ reducing the quality of life.^9^

Aromatase has been detected in the amygdala of mice and humans.^10^, ^11^ The central amygdala produces estrogens through the conversion of testosterone to estrogens by the enzyme aromatase, and these estrogens alter neuronal transmission within the amygdala.^12^ Estrogen receptors localized to aromatase‐producing neurons within the amygdala suggest estrogens can affect aromatase expression.^13^ Consistent with this idea, sex hormones alter gene expression within the central amygdala of rats injected with VZV.^14^ Estrogens also reduce VZV‐associated pain.^15^, ^16^, ^17^ This reduction in VZV‐associated orofacial pain involves GABA release in the lateral parabrachial.^18^ It is likely release of GABA within the lateral parabrachial inhibits neurons that transmit ascending orofacial pain signals from the spinal trigeminal nucleus and trigeminal nucleus caudalis.^19^, ^20^

Based on the previous work, it was hypothesized that estrogens from the central amygdala attenuate VZV‐induced orofacial pain. To address this question, aromatase and estrogen receptor alpha (ERα) agonist were infused into the central amygdala after injecting the whisker pad of rats with VZV. Estradiol concentration was measured in the central amygdala. Pain and GABA release in the lateral parabrachial and neuronal activity in the lateral parabrachial, trigeminal nucleus caudalis, and trigeminal ganglia were then quantitated after infusion.

## 2 MATERIALS AND METHODS

### 2.1 Animal husbandry

Animal husbandry and animal welfare were in accordance with the Institutional Animal Care and Use Committee Guidebook, Texas A&M University College of Dentistry Institutional Animal Care and Use Committee. Procedures and protocols were approved by the Texas A&M University College of Dentistry Institutional Animal Care and Use Committee. Adult male Long–Evans rats (300 g) were bred in‐house and kept on a 14:10 light/dark cycle. The rats were given food and water ad libitum.

### 2.2 Treatment and experimental groups

Sixty‐four rats had a guide cannula surgically placed in the central amygdala bilaterally. An engineered GABA sensor viral construct was infused in the right lateral parabrachial. Then, a fiberoptic filament was inserted in the right lateral parabrachial. The rats were allowed 4 weeks to recover from surgery. Rats were divided, and half received an injection of 100 μL of MeWo cells infected with VZV (>50,000–60,000 pfu/μL) or the same volume of control MeWo cells (human skin cell line) lacking virus into the left whisker pad. Starting 1 week after whisker pad injection, the animals were divided, receiving either a 1 μL bilateral infusion of 5 mg/mL letrozole or a 1 μL infusion of 10 μg/mL ERα agonist (4, 4′, 4″‐(4‐Propyl‐[1*H*]‐pyrazole‐1,3,5‐triyl)*tris*phenol) (PPT) or vehicle 3 h before behavioral testing. Dimethyl sulfoxide (DMSO) 99% was injected as a vehicle. The drug dosage and testing 3 h after administering the drug produced the maximal behavioral response in prior studies.^16^ The groups were no VZV/no letrozole, no VZV/letrozole, VZV/no letrozole, VZV/letrozole or no VZV/no PPT, no VZV/PPT, VZV/no PPT, and VZV/PPT. Note that the no‐letrozole and no‐PPT descriptors in the treatment groups refer to vehicle administration, and the no‐VZV descriptor refers to administering MeWo control cells without virus. After behavioral testing, the 64 rats were sacrificed and the brain was perfused with paraformaldehyde for immunofluorescent studies. In a separate experiment, 48 rats (six rats per group) were cannulated infused with letrozole or PPT and injected with VZV, but the brain was frozen and the tissue was isolated for ELISA. A total of 112 rats were used in these studies.

### 2.3 Guide cannula and fiberoptic filament surgery

Rats (300 grams) underwent surgery to place two 23‐gauge guide cannulas into the central amygdala bilaterally. In this surgery, the rats were anesthetized with 2% isoflurane with an airflow of 2 L/min. Using sterile technique, the guide cannulas (C313G Plastics One, Roanoke, VA) were stereotaxically placed using the coordinates 2.4 mm posterior of Bregma and 4.2 mm from midline at a depth of 8.4 mm from the top of the skull. The cannulas were held in place with four stainless steel screws placed within the skull and dental cement (Metabond, Parkell Inc., Edgewood, NY). The guide cannulas were closed with obturators. In this same surgery, the right lateral parabrachial was infused with 1 μL AAV1 pAAV.hSynap.iGABASnFR (Addgene, Watertown, MA) at stereotaxic coordinates 0.0 mm from Lambda and 2.4 mm from midline and at a depth of 6.6 mm, flat skull. A Stoelting stereotaxic syringe pump system infused at a rate of 50 nL/min. After infusion, the needle was left in place for 5 min and then removed. A single, clear borosilicate glass fiber 9.0 mm in length by 0.43 mm wide made by Doric Lenses (Quebec Canada, MFC_400/430‐0.66_9mm_MF1.25_FLT) was placed on the right side lateral parabrachial at coordinates 0.0 mm from Lambda and 2.4 mm from midline at a depth of 6.5 mm. The lens was held in place with four stainless steel screws placed within the skull and dental cement. After surgery, the animals received buprenorphine 30 μg/kg subcutaneously.

### 2.4 Infusion protocol

Infusion of the central amygdala required the removal of the obturators and the insertion of an injection syringe that projected 0.5 mm below the guide cannula. Bilateral (1 μL) infusions included letrozole (5 mg/mL) or PPT (10 μM) or vehicle DMSO.^21^, ^22^ Patients having a letrozole plasma concentration of 2.5 mg/mL showed specificity for aromatase with no effect on adrenal hormones,^23^, ^24^ and a 5‐mg dose was even more effective with no side effects.^25^, ^26^ PPT is specific for ERα and, at a concentration of 1 microgram, has a 410‐fold higher affinity for ERα versus ERβ.^27^ This amount of infusion volume results in localization to the central amygdala region.^18^

### 2.5 Fluorescence data capture and analysis

Fluorescence from the glass fiber in the lateral parabrachial was captured during behavioral testing using a Tucker‐Davis Technologies instrument (RZ10X) and Synapse software (Alachua, FL). During fluorescent measurement, the parabrachial was excited at 465 nm using an LED light. Simultaneously, a 405‐nm LED light was used as an isobestic fluorescent signal to measure motion artifacts. The Δ*F*/*F* of the two signals was calculated using Python software, and the area under the curve for the positive Δ*F*/*F* peaks was analyzed with Prism 7.05 (GraphPad Software, La Jolla, CA). Positive peaks less than 10% of the baseline signal were excluded.

Specifically, the iGABASnFR fluorescent signal was calculated using a least‐squares regression line between the 465‐nm fluorescence signal (dependent variable) and the 405‐nm fluorescence signal (independent variable).^28^ Residuals resulting from the prediction of the 465‐nm signal using the 405‐nm signal were divided by the predicted 465‐nm signal giving the Δ*F*/*F*. Effectively, these are regression residuals normalized by the corresponding estimated values. Since regression tends to minimize any deviations from the estimated line, values within the −0.5 to 0.5 s peri‐stimulus window were excluded from line‐fitting to avoid reduction of potential Δ*F*/*F* signal due to the stimulus. Pre‐ (−0.5–0.0 s) and post‐stimulus (0.0–0.5 s) areas under the curve (AUCs) were calculated from Δ*F*/*F* time series values. Their difference was used as an effect in treatment comparisons.

### 2.6 Behavioral testing

Place Escape/Avoidance Paradigm (PEAP) testing was performed 3 h after infusing the drug to determine pain. To accomplish this, the rats were placed in a 30 cm × 30 cm × 30 cm acrylic box where half the box was covered in black cloth. This test chamber was modeled from the PEAP test performed by Fuchs's laboratory.^29^ This assay was used to measure the motivation/affective aspect of pain.^29^, ^30^ The PEAP test is based on the assumption that if animals escape and/or avoid a noxious stimulus, then the stimulus is aversive to the animal. Rodents being nocturnal in nature preferred to stay on the dark side when placed into the test chamber that has a light and dark side. After placing the rat in the test chamber, the rat was immediately poked with a 60‐g filament every 15 s on the injected side if the rat was on the dark side and, on the non‐injected side, if it was on the light side. The target region for the poking was the area below the eye and caudal to the whisker pad. This region is innervated by the second branch of the trigeminal ganglion,^31^ the nerve infected by VZV injection of the whisker pad. The time spent on the dark side of the box was recorded in 5‐min bins, and testing was performed for a total of 30 min. The theory behind the test is that if the rat is experiencing VZV‐induced pain when poked in the sensitive area, it will not stay on its preferred dark side but will move to the non‐preferred light side and stay there to avoid the pain poke.

### 2.7 Immunofluorescent staining

After behavioral testing, the rats were given anesthesia consisting of 100 mg/kg ketamine and 10 mg/kg xylazine. The animals were then perfused with PBS followed by 4% paraformaldehyde. Fixed tissues were stored in 25% sucrose, frozen, and cryo‐sectioned, and the 32‐μm sections placed on Histobond slides (VWR international, Radnor, PA). The tissue was then blocked with a PBS solution containing 5% normal goat serum (Sigma‐Aldrich, St. Louis, MO) and 0.3% Triton X‐100 for 2 h at room temperature. The slides were then incubated in a primary antibody solution overnight at 4°C. The primary antibodies consisted of a mixture of the mouse monoclonal NeuN antibody (Millipore, Billerica, MA, MAB377) at a 1:150 dilution and rabbit c‐Fos antibody (Cell Signaling, Danvers, MA, 9F6) at a 1:150 dilution. The primary antibodies were diluted with PBS, 5% bovine serum albumin, and 0.3% Triton X‐100. After incubation with the primary antibodies, the slides were then rinsed three times in PBS and Triton X 100 for a total of 45 min and placed for 2 h in secondary antibody and PBS and 0.3% Triton X‐100. Secondary antibodies (1:500 dilution) included a mixture of goat anti‐mouse 568 and goat anti‐rabbit 488 (Invitrogen, Carlsbad, CA). After rinsing the slides three times in PBS for a total of 45 min, the slides were mounted with Fluoromount‐G mounting medium containing Hoechst 33342 stain (Electron Microscopy Sciences, Hatfield, PA). The fluorescent signal was imaged using a Nikon fluorescent microscope mounted with a Photometrics CoolSnap K4 CCD camera (Roper Scientific, Inc., Duluth, GA) and NIS‐Elements imaging software.

Cell counts were completed by a reviewer blinded to the treatment groups. The injection site was the center point from which sections were selected. Every other section was selected for staining. Typically, three sections were counted for each animal. The slides were analyzed using ImageJ software; the average background for the slides within a treatment group was subtracted from the image, and a fluorescent signal associated with a cell nucleus was counted as a positive cell. Counts were completed for the number of NeuN/c‐Fos and total NeuN‐stained cells within a 0.125‐mm^2^ field adjacent to the injection site. On each section, two randomly selected fields near the tip of the injection site were counted. Counts were completed within the lateral parabrachial nucleus, trigeminal nucleus caudalis, and trigeminal ganglia, and cell counts from the two fields on each section were then averaged. This average count for the three sections was averaged for each animal. Values were given as a mean and standard error of the mean (SEM) representing an average of the values for the animals in each treatment group. Six of the eight rats in each group were perfused, and one animal was eliminated from the count due to the loss of tissue.

### 2.8 Determination of estradiol concentration within the central amygdala

A group of rats were cannulated bilaterally and then 4 weeks later bilaterally injected with VZV or control. One week after injection, the animals were infused bilaterally with letrozole or PPT, and the animals were sacrificed 3 h after infusion. The brain was chilled on dry ice and coronally sectioned with a brain slicer to create 2‐mm‐thick sections (Zivic Instruments, Pittsburg, PA), and the CeA region was isolated by 2‐mm punch biopsy.^14^ The amount of estradiol was quantitated in the tissue punches by ELISA using a kit (Cat. #EKF57979) from Biomatik in Wilmington, DE. Total protein was quantitated in each punch using the BCA assay (Thermo Fisher Scientific).

### 2.9 Statistics

Data were tested with the KS normality test (*α* = .05). PEAP data were passed the normality test and were analyzed by three‐way ANOVA. The three variables were the time spent on the light or dark side of the box (collected in six time bins, i.e., 5, 10, 15, 20, 25, and 30 min), virus treatment (i.e., VZV), and drug treatment (either letrozole or PPT). Cell count and ELISA data were normal and were analyzed by two‐way ANOVA. The independent variable was cell counts, and the dependent variable was treatment (i.e., VZV injection and drug infusion). When a significant effect was observed, Tukey's post hoc tests were completed. GABA release data were analyzed with the non‐parametric Mann–Whitney test as not all the data were distributed normally (Prism 7.05, GraphPad Software, La Jolla, CA).

## 3 RESULTS

### 3.1 Enhanced pain after letrozole treatment

**FIGURE 1:**
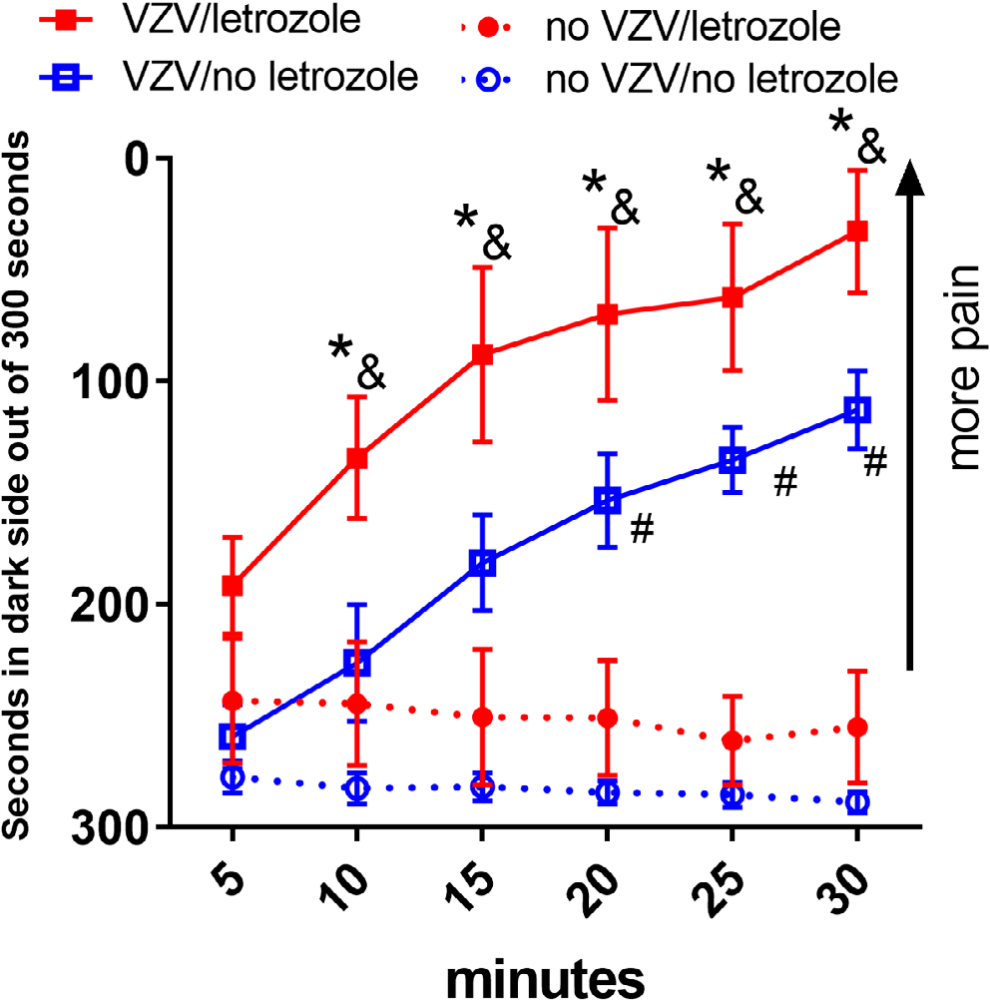
Infusion of aromatase inhibitor letrozole into the central amygdala increased VZV‐induced pain. The hashtag symbol indicates a significant difference (*p* < .05) between the no‐VZV/no‐letrozole group (open blue circles) and VZV/no‐letrozole group (solid blue squares). The ampersand symbol indicates a significant difference (*p* < .05) between the no‐VZV/letrozole group (red open circles) and the VZV/letrozole group (solid red squares). The asterisks indicate a significant difference (*p* < .05) between the VZV/no letrozole (solid blue squares) and VZV/letrozole group (solid red squares). There were eight animals per group. Values are the mean and SEM.

Injecting the whisker pad with VZV significantly increased the pain response *F*(1, 168) = 187, *p* < .0001. Infusion of letrozole into the central amygdala significantly increased the pain response *F*(1,168) = 31, *p* < .0001 (Figure 1, compare red solid squares to the blue solid squares). The interaction between VZV and letrozole was significant *F*(1, 168) = 5, *p* = .02. The interaction between drug and time was significant *F*(5, 168) = 3.8, *p* < .01. The interaction between VZV and time was not significant *F*(5, 168) = 0.11, *p* = .9. Animals that received no VZV remained on the dark side throughout most of the testing period (Figure 1, open red and open blue circles).

### 3.2 Infusion of ERα agonist PPT into the central amygdala affects pain

**FIGURE 2:**
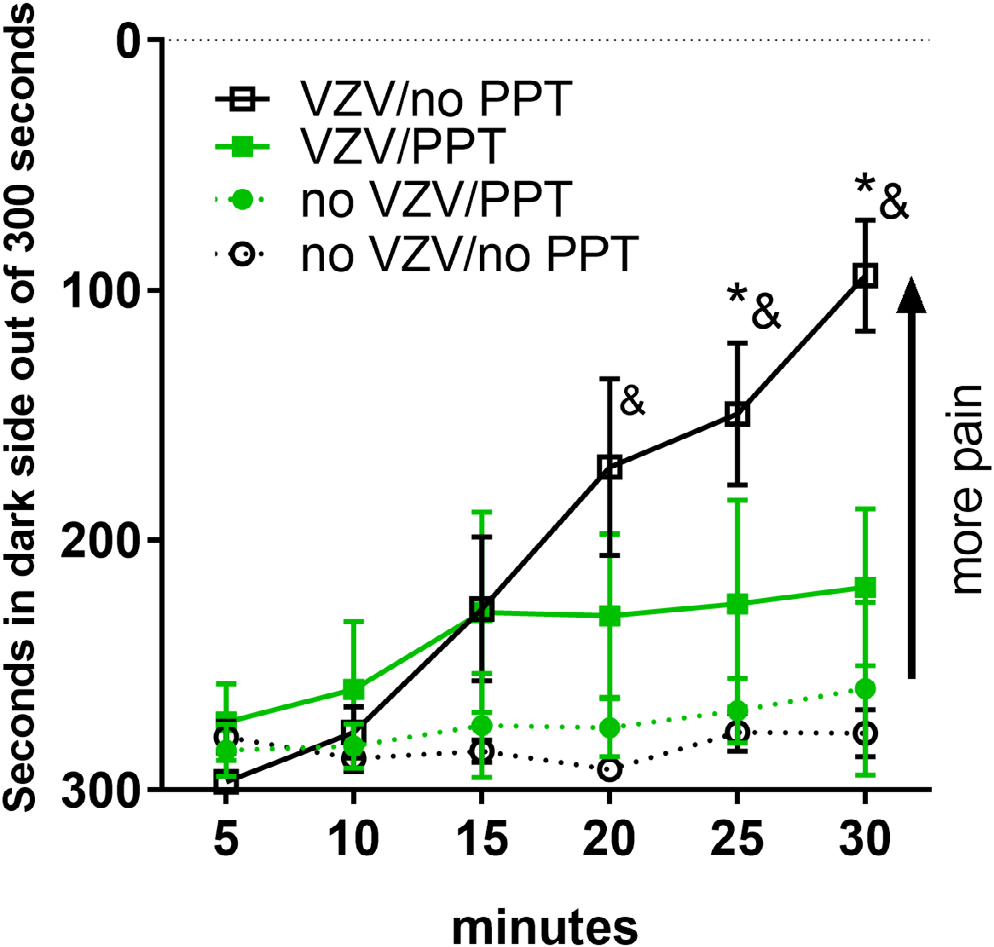
Infusion of the ERα agonist PPT reduced the VZV‐induced pain response. The ampersand symbol indicates a significant difference between the VZV/no‐PPT group (open black squares) and the no‐VZV/no‐PPT group (open black circles). The asterisks indicate a significant difference (*p* < .05) between the VZV/no‐PPT group (open black squares) and VZV/PPT group (solid green squares). There were eight animals per group. Values are the mean and SEM.

Infusing ERα agonist PPT into the central amygdala significantly reduced the pain response. PPT infusion significantly reduced the pain response in rats after VZV injection *F*(1, 168) = 38, *p* < .0001 (Figure 2, compare black open squares to the solid green squares). There was a significant increase in pain resulting from VZV injection in the group that received no PPT (Figure 2, compare black open squares to the black open circles). VZV injection did not significantly increase the pain response after infusing PPT *F*(1, 168) = 0.8, *p* = .36 (Figure 2, compare solid green squares to solid green circles). There was a significant interaction between VZV injection and PPT infusion *F*(1, 168) = 3.0, *p* = .05. There was a significant interaction between PPT infusion and time *F*(5, 168) = 3.8, *p* < .01. There was no significant interaction between VZV injection and time *F*(5, 168) = 1.2, *p* = .2.

### 3.3 GABA release within the lateral parabrachial nucleus after letrozole administration

**FIGURE 3:**
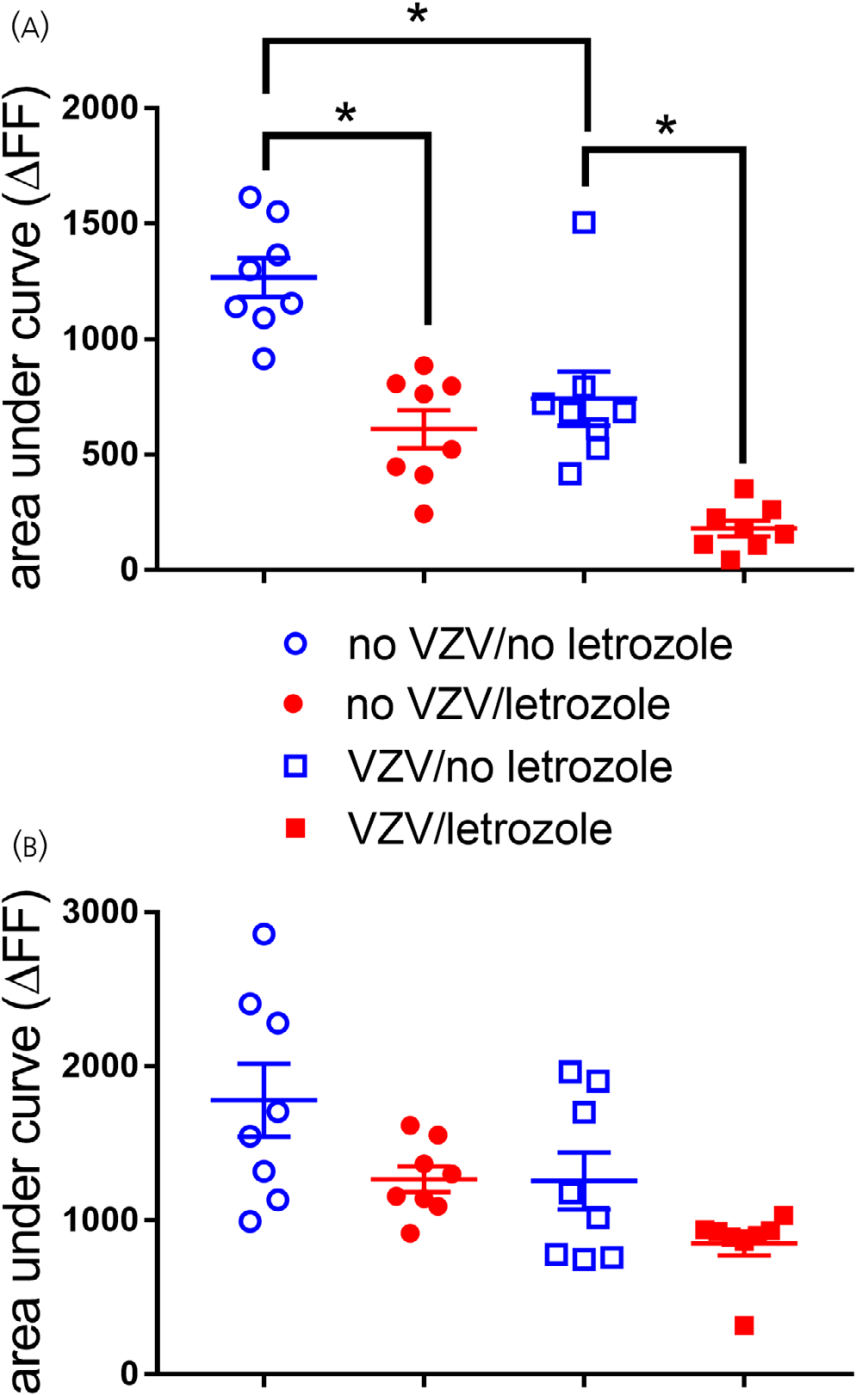
GABA release declines in the lateral parabrachial after infusion of letrozole in the central amygdala. The histograms show the fluorescent signal within the lateral parabrachial for an engineered receptor (iGABASnFR) that binds GABA in rats that were injected in the whisker pad with MeWo cells containing VZV or MeWo cells only (no VZV) and then infused with letrozole (5 mg/mL) or no letrozole (i.e., DMSO) into the central amygdala. Panel A represents spontaneous GABA release, and panel B represents GABA release during a poke to the whisker pad. There were eight animals per group. Each point on the histogram represents an individual animal, and the asterisk indicates *p* < .05.

Neurons within the central amygdala project axons to the lateral parabrachial and the terminals of these axons then release GABA into the lateral parabrachial.^18^ GABA release in the lateral parabrachial was measured during the behavioral pain testing 3 h after infusing the central amygdala with letrozole or vehicle. During the testing, the whisker pad was poked every 15 s. GABA release was measured during a poke to the face, termed evoked, and between each poke, termed spontaneous GABA release. On average, rats given no VZV with no letrozole (Mdn = 1228) had more spontaneous GABA release compared to rats treated with no VZV and letrozole (Mdn = 643). This difference was statistically significant, *U*(*N* = 8) =0, *p* = .0002 (Figure 3A, compare blue open circles to red open circles). The VZV group with no letrozole (Mdn = 684) had significantly more spontaneous GABA release, *U*(*N* = 8) =0, *p* = .0002, than the VZV group with letrozole (Mdn = 169) (Figure 3A, compare solid blue squares to the solid red squares). VZV did significantly reduce spontaneous GABA release in rats without letrozole (Figure 3A, compare blue open circles to blue solid squares).

Rats given no VZV with no letrozole (Mdn = 1626) had more evoked GABA release compared to rats with no VZV and letrozole (Mdn = 1228), but this difference was not significant, *U*(*N* = 8) =22, *p* = .16 (Figure 3B). VZV‐injected rats with no letrozole (Mdn = 1099) had more evoked GABA release than the VZV group with letrozole (*Mdn* = 915), but this difference was not significant, *U*(*N* = 8) =0, *p* = .32 (Figure 3B).

### 3.4 Infusion of ERα agonist increased GABA release

**FIGURE 4:**
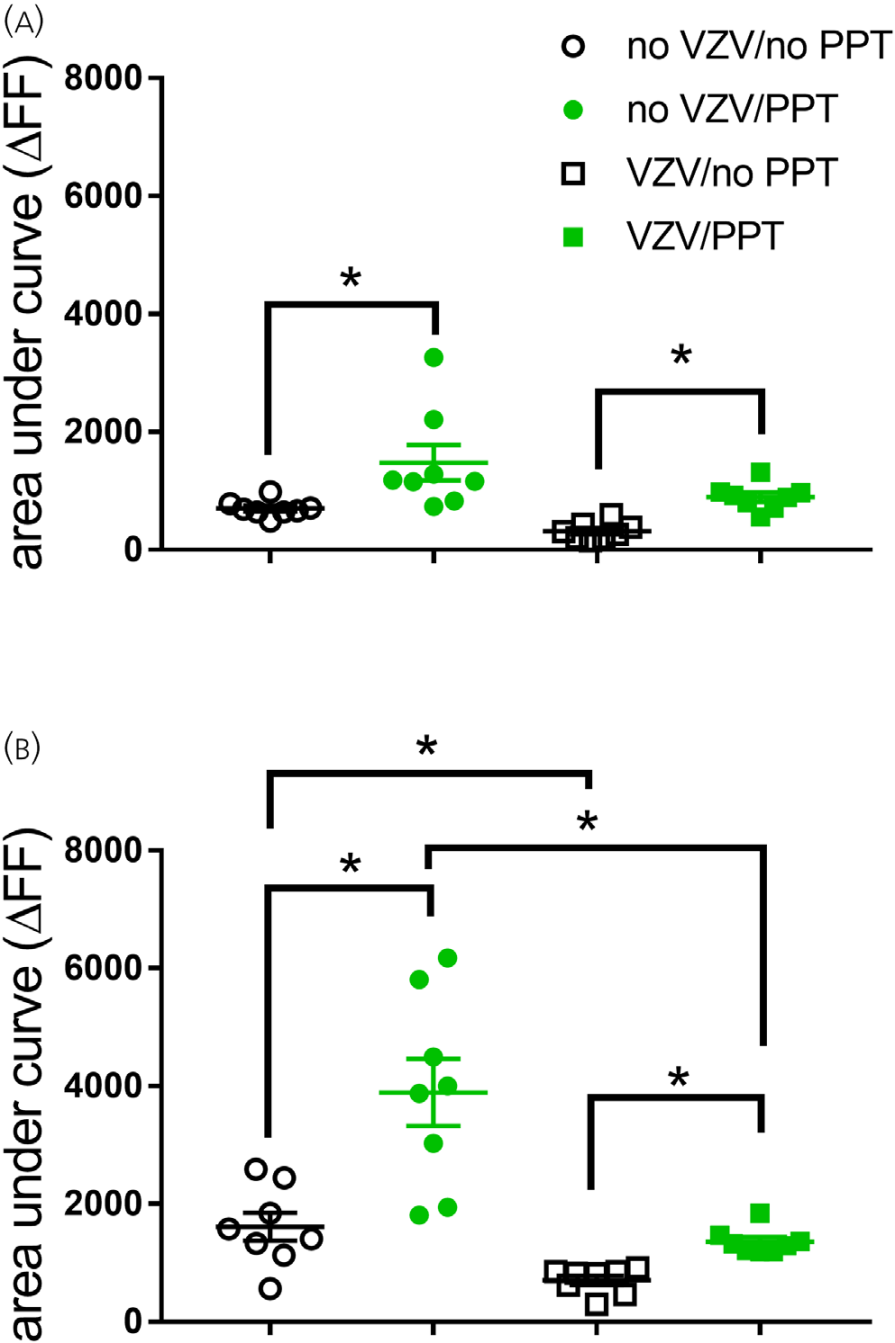
GABA release increases in the lateral parabrachial region after infusion of PPT in the central amygdala. The histograms show the fluorescent signal for an engineered receptor that binds GABA (iGABASnFR) in rats treated with and without VZV and PPT. Panel A represents spontaneous GABA release, and panel B represents GABA release during a poke to the whisker pad. There were six animals per group. Each point on the histogram represents an individual animal, and the asterisk indicates *p* < .05.

Spontaneous GABA release in the no‐VZV and no‐PPT groups (Mdn = 675) was less than in the no‐VZV and PPT groups (Mdn = 1174). This difference was statistically significant, *U*(*N* = 8) = 3, *p* = .001 (Figure 4A, compare black open circles to solid green circles). Giving VZV with no PPT (Mdn = 285) induced significantly less spontaneous GABA release, *U*(*N* = 8) = 1, *p* = .0003, as compared to rats treated with VZV and PPT (Mdn = 904).

Evoked GABA release in rats given no VZV with no PPT (Mdn = 1493) was reduced as compared to rats with no VZV and PPT (Mdn = 3940). This difference was significant, *U*(*N* = 8) =5, *p* = .003 (Figure 4B). VZV‐injected rats with no PPT (Mdn = 821) had a significantly reduced evoked GABA release as compared to VZV‐injected rats given PPT (*Mdn* = 1307), and this difference was significant, *U*(*N* = 8) =0, *p* = .0002 (Figure 4B). VZV injection decreased evoked GABA release; comparing the no‐VZV with no‐PPT groups to the VZV with no‐PPT groups, *U*(*N* = 8) =6, *p* = .005 (Figure 4B, compare the open black circles to the open black squares). Also, we compare the no VZV with PPT group to the VZV with PPT group, *U*(*N* = 8) = 1, *p* = .003 (Figure 4B, compare the solid green circles to the solid green squares).

### 3.5 Neuronal activity in orofacial pain pathway

**FIGURE 5:**
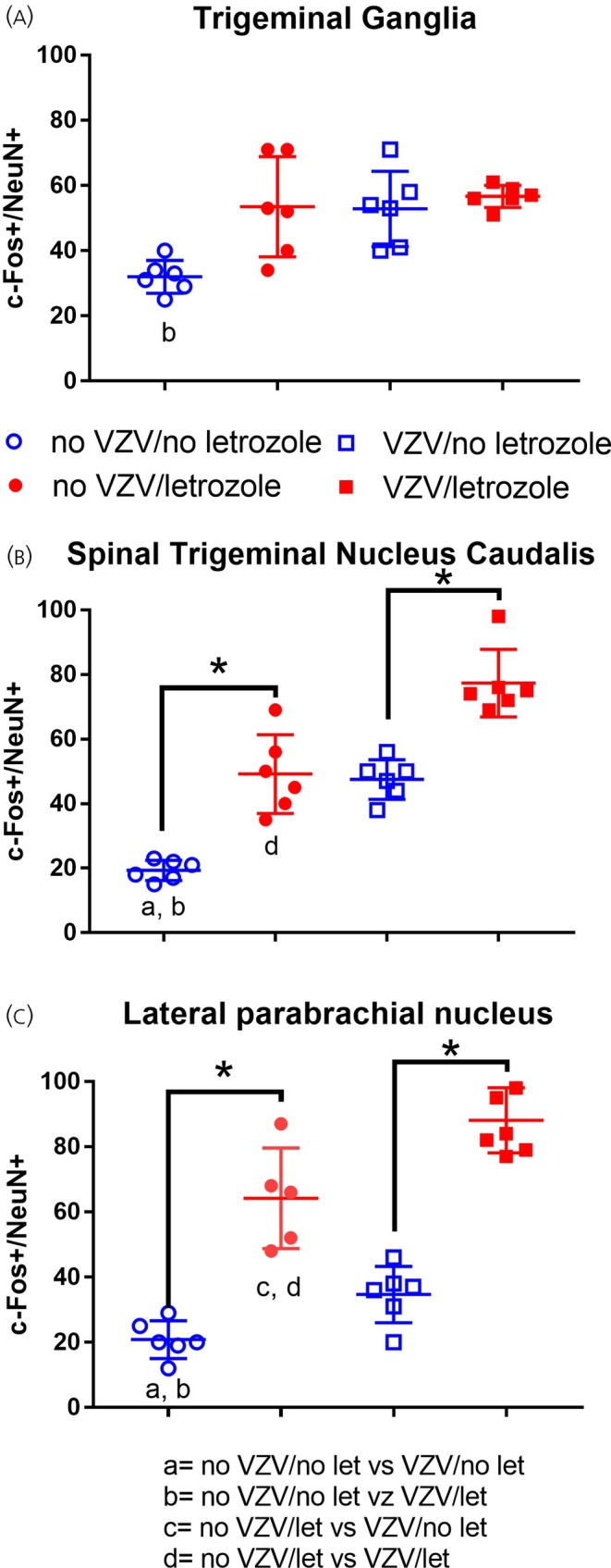
NeuN and c‐Fos‐positive cells within the trigeminal ganglia, spinal trigeminal nucleus caudalis, and lateral parabrachial. Rats were injected in the whisker pad with MeWo cells containing VZV or MeWo cells only (no VZV) and then infused with letrozole (5 mg/mL) or no letrozole (i.e., DMSO) into the central amygdala. The asterisks indicate a significant difference of *p* < .05. The letter “a” indicates a significant difference (*p* < .05) between the no VZV/no letrozole (open blue circles) and the VZV/no letrozole (solid blue squares). The letter “b” indicates a significant difference (*p* < .05) between the no VZV/no letrozole (blue open circles) and VZV/letrozole groups (solid red squares). The letter “c” indicates a significant difference (*p* < .05) between the no‐VZV/letrozole group (open red circles) and the VZV/no‐letrozole group (solid blue squares). The letter “d” indicates an significant difference (*p* < .05) between no‐VZV/letrozole group (open red circles) and the VZV/letrozole group (solid red squares). There were six animals per group. Each point represents an animal. Values are the mean and SEM.

GABA is released into the lateral parabrachial, and this GABA blocks orofacial pain signals from the face that are ascending through the spinal trigeminal nucleus and trigeminal nucleus caudalis.^19^, ^20^ Thus, neuronal activity was measured in the trigeminal ganglia, trigeminal nucleus caudalis, and the lateral parabrachial after both letrozole and PPT administration. The number of c‐Fos‐positive cells did not increase significantly in the trigeminal ganglia with letrozole treatment (Figure 5A). Letrozole did significantly increase the number of cells co‐localized for c‐Fos and NeuN in the trigeminal nucleus caudalis (Figure 5B) and the lateral parabrachial (Figure 5C). In the trigeminal ganglia, there was a significant effect for VZV *F*(1, 20) = 8.3, *p* < .01, but not for letrozole F(1, 20) = 2.2, *p* = .14, with no significant interaction *F*(1, 20) = 0.8, *p* = .36. In the trigeminal nucleus caudalis, there was a significant effect for VZV *F*(1, 20) = 70, *p* < .0001, and letrozole *F*(1, 20) = 62, *p* < .0001, with no significant interaction F(1, 20) = 0, *p* = .99. In the lateral parabrachial, there was a significant effect for VZV *F*(1, 19) = 135, *p* < .0001, and letrozole F(1, 19) = 22, *p* < .001, with no significant interaction *F*(1, 19) = 1.9, *p* < .17.

**FIGURE 6:**
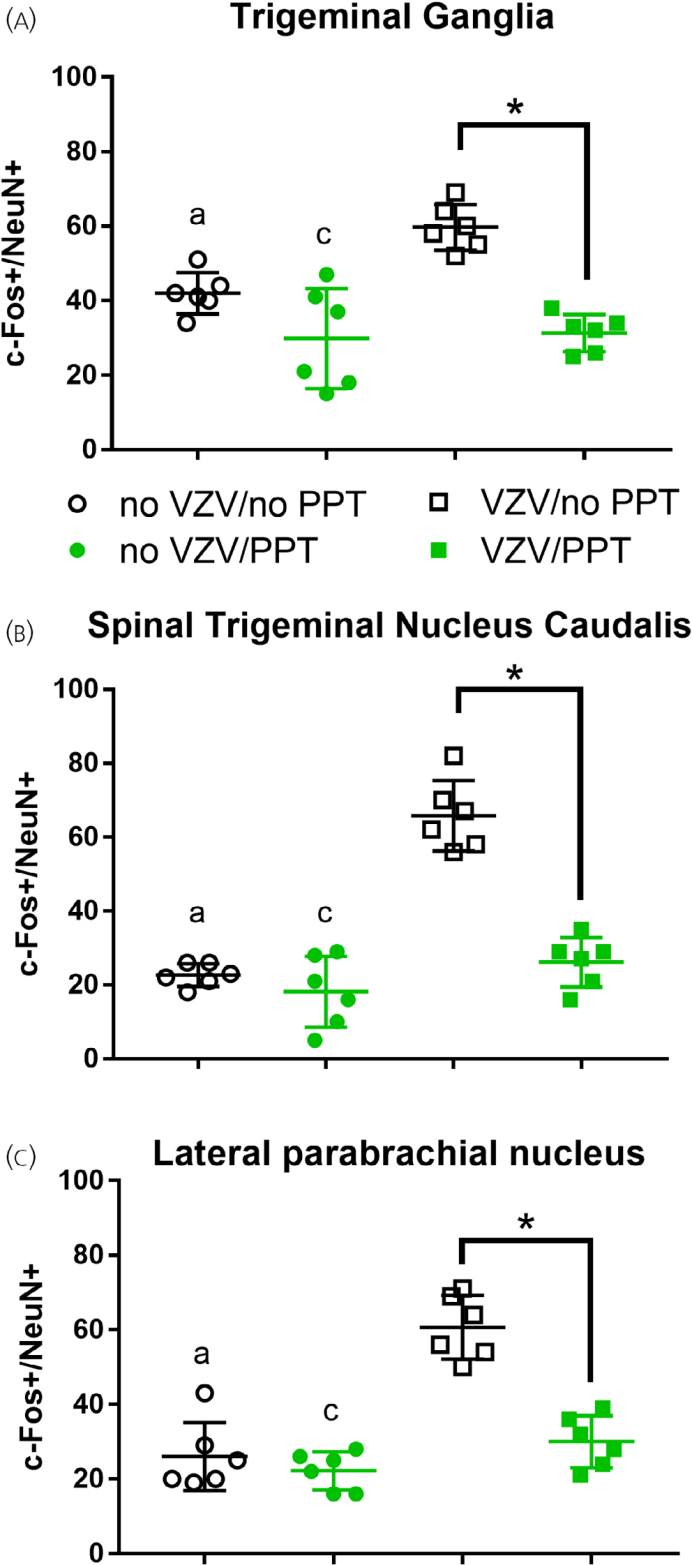
Cell counts within the trigeminal ganglia, spinal trigeminal nucleus caudalis, and lateral parabrachial region for cells stained for NeuN and c‐Fos after injecting VZV or no VZV into the whisker pad and then infusing PPT (10 μg/mL) or no PPT (i.e., DMSO) into the central amygdala. The asterisks indicate a significant difference of *p* < .05. The letter “a” indicates a significant difference (*p* < .05) between the no VZV/no PPT (open black circles) and VZV/no PPT (open blue squares). The letter “c” indicates a significant difference (*p* < .05) between the no‐VZV/PPT group (solid green circles) and the VZV/no‐PPT group (open black squares). There were six animals per group. Each point represents an animal. Values are the mean and SEM.

PPT significantly reduced the number of c‐Fos cells in the trigeminal ganglia (Figure 6A), trigeminal nucleus caudalis (Figure 6B), and the lateral parabrachial (Figure 6C). In the trigeminal ganglia, there was a significant effect for VZV *F*(1, 20) = 35.7, *p* < .0001, and PPT *F*(1, 20) = 8.0, *p* < .05, with a significant interaction *F*(1, 20) = 5.7, *p* < .05. In the trigeminal nucleus caudalis, there was a significant effect for VZV *F*(1, 20) = 49.1, *p* < .0001, and PPT *F*(1, 20) = 65.9, *p* < .0001, with a significant interaction *F*(1, 20) = 31.1, *p* < .0001. In the lateral parabrachial, there was a significant effect for VZV *F*(1, 20) = 30.7, p < .0001, and PPT *F*(1, 20) = 46.6, *p* < .0001, with a significant interaction *F*(1, 20) = 18.5, *p* < .001. The total number of NeuN‐positive cells was not significantly different between treatment groups (data not shown).

**FIGURE 7:**
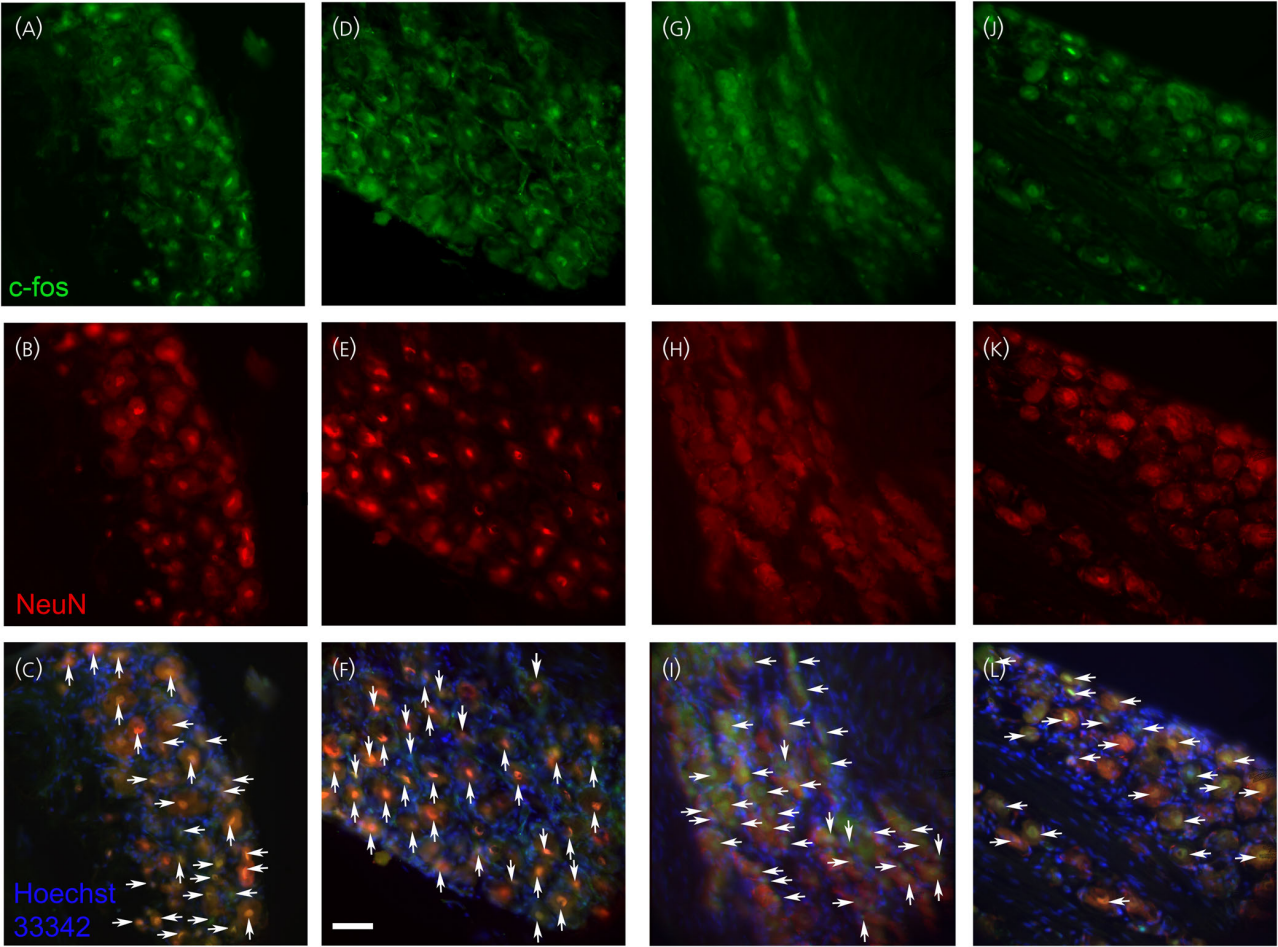
NeuN and c‐Fos‐stained cells within the trigeminal ganglia. c‐Fos‐stained cells (green, panels A, D, G, and J) and NeuN‐positive cells (red, panels B, E, H, and K) and cells that co‐localize for c‐Fos and NeuN are shown (yellow, panels C, F, I, and L, arrows). Images represent a rat treated with VZV and no letrozole (panels A‐C), a rat treated with VZV and letrozole (panels D–F), a rat treated with VZV and no PPT (panels G–I), and a rat treated with VZV and PPT (panels J–L). Nuclei are stained with Hoechst 33342 in panels C, F, I, and L. Bar = 100 μm.

Images of sections taken from representative rats show the number of c‐Fos‐positive neurons in the trigeminal ganglia increased after letrozole treatment, comparing the VZV no‐letrozole group (Figure 7A–C) to the VZV with letrozole group (Figure 7D–F). The number of immunofluorescent positive c‐Fos cells decreased with PPT administration in the trigeminal ganglia, comparing the VZV no‐PPT group (Figure 7G–I) to the VZV with PPT group (Figure 7J–L).

**FIGURE 8:**
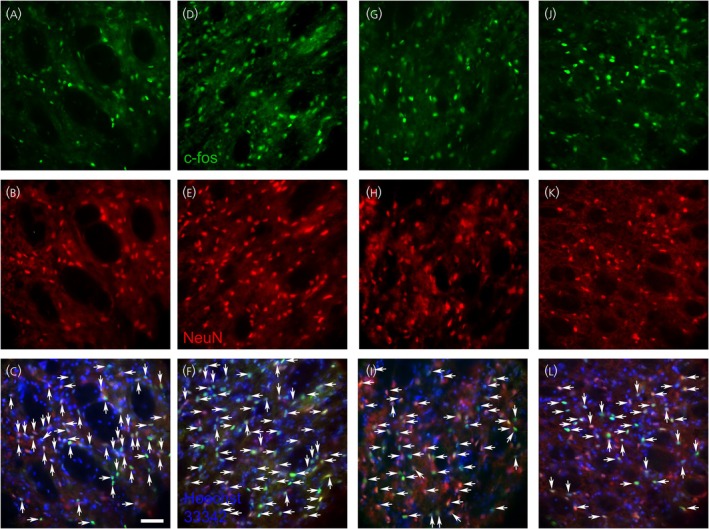
NeuN and c‐Fos‐stained cells within the trigeminal nucleus caudalis. c‐Fos‐stained cells (green, panels A, D, G, and J) and NeuN‐positive cells (red, panels B, E, H, and K) and cell that co‐localize for c‐Fos and NeuN are shown (yellow, panels C, F, I, and L, arrows). Images are from a representative rat treated with VZV and no letrozole (panels A–C), a rat treated with VZV and letrozole (panels D–F), a rat treated with VZV and no PPT (panels G–I), and a rat treated with VZV and PPT (panels J–L). Nuclei are stained with Hoechst 33342 in panels C, F, I, and L. Bar = 100 micrometers.

Letrozole treatment increased the number of c‐Fos‐positive neurons in the trigeminal nucleus caudalis, comparing the VZV no‐letrozole group (Figure 8A–C) to the VZV with letrozole group (Figure 8D–F). PPT treatment decreased the number of c‐Fos‐positive neurons in the trigeminal nucleus caudalis, comparing the VZV no‐PPT group (Figure 8G–I) to the VZV with PPT group (Figure 8J–L).

**FIGURE 9:**
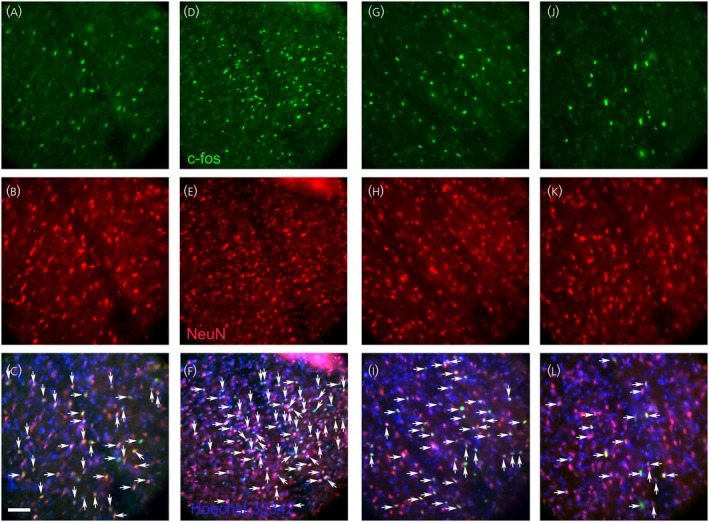
NeuN and c‐Fos‐stained cells within the lateral parabrachial nucleus. c‐Fos‐stained cells (green, panels A, D, G, and J) and NeuN (red, panels B, E, H, and K) and cell that co‐localize for c‐Fos and NeuN are shown (yellow, panels C, F, I, and L, arrows). Images show a representative rat treated with VZV and no letrozole (panels A–C), a rat treated with VZV and letrozole (panels D–F), a rat treated with VZV and no PPT (panels G–I), and a rat treated with VZV and PPT (panels J–L). Nuclei are stained with Hoechst 33342 in panels C, F, I, and L. Bar = 100 μm.

In the lateral parabrachial, we compare the VZV no‐letrozole group (Figure 9A–C) with the VZV with letrozole group (Figure 9D–F); letrozole treatment increased the number of c‐Fos‐positive neurons. In the lateral parabrachial, compare the VZV no‐PPT group (Figure 9G–I) to the VZV with PPT group (Figure 9J–L), PPT treatment decreased the number of c‐Fos positive neurons.

### 3.6 Letrozole reduced estradiol in the central amygdala

**FIGURE 10:**
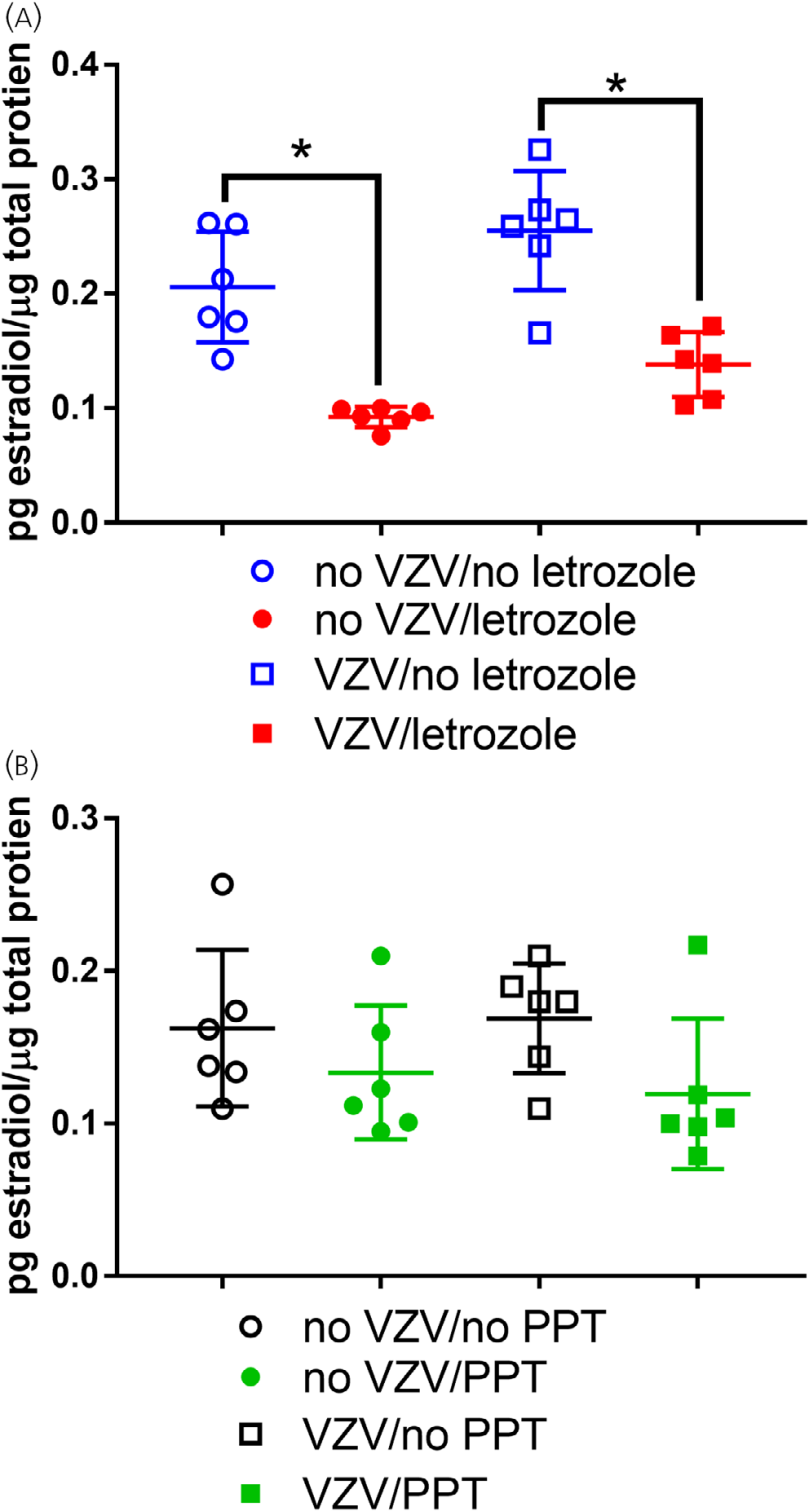
Estradiol concentration within the central amygdala after infusion of either letrozole or estrogen receptor agonist PPT. The amount of estradiol was reported as the amount of estradiol for a total weight of protein within a tissue plug taken from the central amygdala after VZV injection and drug infusion. The amount of estradiol was quantitated after infusing the central amygdala with letrozole (panel A) or ERα agonist PPT (panel B). There were six animals in each treatment group; each point on the graph represents a different animal. The asterisks indicate a significant difference of *p* < .05. Values are the mean and SEM.

The estradiol concentration within the central amygdala decreased after letrozole treatment *F*(1, 20) = 27.9, *p* < .0001 (Figure 10A). VZV injection had a small main effect *F*(1, 20) = 5.6, *p* = .03, but post hoc tests were not significant, and no interaction was detected *F*(1, 20) = 0.06, *p* = .8. A decrease in estradiol was measured after PPT treatment *F*(1, 20) = 4.4, *p* < .05, but post hoc tests and the interaction were not significant (Figure 10B).

## 4 DISCUSSION

Based on the previous work, our laboratory hypothesized that estrogens produced within the central amygdala attenuate VZV‐induced orofacial pain. To address this question, our laboratory reduced aromatase activity with letrozole and administered an ERα agonist PPT into the central amygdala of rats injected with VZV. Injection of the whisker pad with VZV increased pain, and letrozole increased the pain response further, while the ERα agonist PPT decreased the pain response. During the behavioral pain test, GABA release decreased after infusing letrozole and increased after infusing PPT into the central amygdala. Consistent with these results, the number of active neurons in the trigeminal nucleus caudalis increased with letrozole and decreased with PPT. Orofacial pain signals ascend from the face through the trigeminal ganglia and then the trigeminal nucleus caudalis to the lateral parabrachial.^19^, ^20^ The results suggest estrogens produced in the central amygdala reduce VZV‐induced orofacial pain and neuronal activity through an estrogen receptor‐dependent mechanism. This idea is supported by data indicating letrozole treatment reduced estradiol content within the central amygdala that this reduced concentration of estradiol was associated with a decrease in GABA release within the lateral parabrachial, contributing to the increase in pain and neuronal activity within the orofacial pain pathway.

Aromatase has been detected in the amygdala of mice and humans.^10^, ^11^ The aromatase within the central amygdala produces estrogens locally from testosterone.^12^ Estrogen receptors localized to aromatase‐producing neurons within the amygdala suggest that estrogens can affect gene expression in these cells.^13^ Once aromatase is expressed in the amygdala, the enzyme has been associated with sex differences in social behaviors by altering synaptic connections.^32^ Thus, previous studies are consistent with the idea that locally produced estrogens (from aromatase) within the central amygdala bind to estrogen receptors to affect neuronal responses and behavior. Our data support this idea because letrozole infusion both decreased estradiol within the central amygdala and increased the pain response.

Humans report pain after taking aromatase inhibitors.^33^, ^34^ Studies have linked aromatase inhibitors to Sjogren's syndrome, resulting in patients suffering from orofacial arthralgia, dry eye, and dry mouth.^35^, ^36^ Inhibition of aromatase with letrozole will increase the pain response, suggesting that estrogens reduce pain in certain pain models.^37^, ^38^ Consistent with these studies, VZV‐associated pain will increase after letrozole treatment.^16^ Inhibition of aromatase increases mechanical hyperalgesia in animals but has no effect on thermal hyperalgesia.^37^ In this study, we measured the motivational/affective aspect of pain after VZV injection, and in the groups that did not receive VZV, there was no increase in pain nor did letrozole increase the pain by itself (i.e., without VZV). These results suggest that letrozole can increase mechanical hyperalgesia but not motivational/affective responses to pain and that aromatase effects can be varied depending on the response being measured.

Estrogens can modulate pain via the nuclear or membrane receptors.^39^, ^40^, ^41^ Moreover, estradiol attenuates pain through both nuclear and membrane receptors.^42^, ^43^, ^44^ In one example, estrogens altered pain transmission via a membrane‐bound estrogen receptor.^45^ Nuclear receptors ERα and ERβ have been shown to regulate gene expression of voltage‐gated sodium channels; these channels play a vital role in the modulation of pain.^46^ In this study, the use of ERα agonist PPT significantly decreased VZV‐associated orofacial pain, suggesting that estradiol binding within the central amygdala affects neuronal function. ERα agonist PPT reduced pain in this study, which is consistent with a study demonstrating that visceral pain is reduced using estrogen receptor agonists^47^ and other studies showing ERβ agonists alleviate neuropathic pain.^48^, ^49^ Currently, the use of estrogen receptor agonists to treat pain in humans has not been reported.

One neuronal function we measured was GABA release. Most of the neurons in the central amygdala are GABAergic^20^, ^50^ and a portion of these GABAergic neurons descends from the central amygdala to the lateral parabrachial region.^20^, ^51^ Estradiol has been shown to reduce GABA function in the amygdala through an estrogen receptor‐dependent mechanism, and this reduction in GABA affects behavioral changes.^52^, ^53^ Administering estradiol can decrease the expression of GABA receptors GAD1 and GAD2 (i.e., genes that produce GABA) within the amygdala, suggesting estradiol will reduce GABA release and signaling through inhibition of gene expression.^54^ Thus, we measured GABA release in the lateral parabrachial during behavior testing. Future studies will look at the expression of various GABA signaling genes and these genes role in signaling VZV‐associated pain. The evoked release of GABA was higher in every group versus the spontaneous response. It is assumed that von Frey filaments used to evoke a response increased descending inhibitory pathways as reflected by increased GABA release. During the spontaneous response, letrozole treatment decreased GABA release; this trend of decreasing response after letrozole treatment was also detected for the evoked response groups, although the response was not significant. Activation of GABA release with filaments potentially “washed out” or reduced the letrozole inhibitory effect and resulted in this insignificant change.

Letrozole increased activity in the trigeminal nucleus caudalis. Moreover, ERα agonist PPT decreased activity in both the trigeminal nucleus caudalis and trigeminal ganglia. The results are consistent with the idea that estrogens binding to the estrogen receptor within the central amygdala reduce activity of neurons in the trigeminal system. Neurons from the trigeminal ganglia and trigeminal nucleus caudalis project to the lateral parabrachial.^19^ Note that GABA neurons descend from the central amygdala to the lateral parabrachial nucleus.^20^, ^51^ GABA release in the parabrachial could inhibit ascending pain signals from neurons within the trigeminal ganglia and trigeminal nucleus caudalis and reduce the orofacial pain response.

Alternatively, descending inhibitory projections from the amygdala to the locus coeruleus^55^ could affect orofacial pain because the locus coeruleus has descending inhibitory axonal projections to the trigeminal nucleus caudalis that control orofacial pain.^56^ In the event that estrogens activated the descending pathway to the trigeminal system, pain would be attenuated.

Previous studies have suggested that estradiol attenuates orofacial pain,^15^, ^57^, ^58^, ^59^, ^60^ but this is the first study to demonstrate a role for estradiol in the central amygdala to attenuate VZV‐associated pain. Estradiol was produced locally because local administration of an aromatase inhibitor resulted in a heightened pain response. This is an interesting result because increased systemic estradiol has also been associated with an attenuated pain response.^15^, ^16^ Administering estradiol systemically and locally to the amygdala can attenuate anxiety, fear, and pain.^61^ Thus, future testing is needed to determine whether systemic estradiol and estradiol produced from testosterone in the central amygdala both alter the pain response.

A few weaknesses of this study are one, we only studied males. The study of females is necessary, noting the sex differences in orofacial pain.^16^ Some animal models show a sex difference while others do not. For example, administering estradiol to the amygdala increased the response to colorectal distension but does not change the evoked hindpaw response.^62^ Similarly, little sex difference is observed with tooth pain or sore gums,^63^ but sex differences are observed in temporomandibular joint disorders,^47^, ^64^, ^65^ highlighting that the pathway pain signals utilize and the mechanism inducing pain influence potential sex differences. Second, this study only looked at the role of ERα in estradiol signaling, and future studies will focus on the potential role of additional estrogen receptors such as ERβ having a role in the pain response. Third, the mean value for the spontaneous GABA release in the no‐VZV/no‐letrozole group was 1200 ± 100, and the spontaneous GABA release for the no VZV/no PPT was 700 ± 50. Statistical analysis demonstrates that there was a significant difference in the mean values for these two controls. One potential explanation for this difference is that a different lot of virus was used in letrozole versus the PPT experiments. We injected the same titers of virus in both experiments, but if GABASnFR viral infection was greater in the letrozole control group, this higher infection rate would explain why more GABA release was measured in the no‐VZV/no‐letrozole group. Whatever the reason, statistically comparing the letrozole and PPT data would not be viable.

In summary, inhibiting aromatase decreased estradiol and GABA release and increased neuronal activity and orofacial pain. Activation of ERα increased GABA release and decreased neuronal activity and orofacial pain. The results are consistent with the idea that locally produced estrogens bind the estrogen receptor and stimulate increased release of GABA in the lateral parabrachial. This GABA then inhibits neuronal activity of ascending pain neurons attenuating the pain response.

## AUTHOR CONTRIBUTIONS

**Phillip Kramer:** Conceptualization; funding acquisition; formal analysis; project administration; supervision; resources; data curation; writing – review and editing; methodology; writing – original draft; visualization. **Lauren Nguyen:** Methodology; investigation. **Paul R. Kinchington:** Project administration; resources; methodology; conceptualization.

## FUNDING INFORMATION

This study was supported by NIDCR grant DE022129 (PRKramer) and a grant from the 30, 300, 3000 Pain Research Challenge from the University of Pittsburgh Clinical and Translational Science Institute (PRKinchington). PRKinchington also acknowledges support from grant NS064022, NEI core grant EY08098, and unrestricted funds from the Eye & Ear Foundation and Research to Prevent Blindness Inc.

## CONFLICT OF INTEREST STATEMENT

The authors declare no conflicts of interest.

### PEER REVIEW

The peer review history for this article is available at https://www.webofscience.com/api/gateway/wos/peer-review/10.1111/jne.70012.

## ETHICS STATEMENT

All authors agree with the contents of this manuscript.

## Data Availability

The data that support the findings of this study are available from the corresponding author upon reasonable request.
